# Marathon-Induced Cardiac Fatigue: A Review over the Last Decade for the Preservation of the Athletes’ Health

**DOI:** 10.3390/ijerph18168676

**Published:** 2021-08-17

**Authors:** Damien Vitiello, Florent Palacin, Luc Poinsard, Marine Kirsch, Steeve Jouini, Véronique Billat

**Affiliations:** 1 URP3625—Institut des Sciences du Sport-Santé de Paris (I3SP), School of Sport Sciences, Université de Paris, 75015 Paris, France; luc.poinsard@gmail.com (L.P.); marine.kirsch@hotmail.fr (M.K.); jouini.s@hotmail.fr (S.J.); 2 Unité de Biologie Intégrative des Adaptations à l’Exercice, Université Paris-Saclay, Univ Evry, 91000 Evry-Courcouronnes, France; palacinflorent@gmail.com (F.P.); veronique.billat@billatraining.com (V.B.)

**Keywords:** cardiac fatigue, cardiac stress, prevention, marathon, recreational athletes

## Abstract

Aim: To provide a state-of-the-art review of the last 10 years focusing on cardiac fatigue following a marathon. Methods: The PubMed, Bookshelf and Medline databases were queried during a time span of 10 years to identify studies that met the inclusion criteria. Twenty-four studies focusing only on the impact of marathons on the cardiac function and factors involved in cardiac fatigue were included in this review. Results: Sixteen studies focused on the impact of marathons on several biomarkers (e.g., C-reactive protein, cardiac troponin T). Seven studies focused on the left (LV) or right (RV) ventricular function following a marathon and employed cardiac magnetic resonance, echocardiography, myocardial speckle tracking and heart rate variability to analyze global and regional LV or RV mechanics and the impact of the autonomic nervous system on cardiac function. One study focused on serum profiling and its association with cardiac changes after a marathon. Conclusions: This review reported a negligible impact of marathons on LV and RV systolic and contractile function but a negative impact on LV diastolic function in recreational runners. These impairments are often associated with acute damage to the myocardium. Thus, the advice of the present review to athletes is to adapt their training and have a regular medical monitoring to continue to run marathons while preserving their cardiac health.

## 1. Introduction

The beneficial effect of regular physical exercise on heart function is now widely recognized by researchers in the field of physical activity and sport around the world and more generally in society. Among the main beneficial effects are the improvement of the lipid profile, carbohydrate homeostasis, decrease in resting blood pressure, blood coagulation, improvement of myocardial perfusion and an increase in cardiac output [1]. While the function of the heart pump is improved by regular exercise of moderate intensity [2], it was first shown in 1964 that the function of the left ventricle (LV) was reduced after prolonged physical exercise (PPE) [3]. Almost twenty years later, work has shown impaired cardiac function in athletes who have achieved PPE and used the concept of Exercise-Induced Cardiac Fatigue for the first time [4]. This phenomenon is defined as a transient decrease in systolic and diastolic ventricular functions and is sometimes associated with an increase in markers of myocardial degradation (i.e., cardiac troponins I) [5].

Endurance activities have been very popular since the end of the 1990′s. The attraction to life in the great outdoors and the desire to know its limits lead more and more people to practice PPE each year [6]. Among these PPE, there are those of moderate duration such as the half-marathon (i.e., between 1–2 h of effort) and the marathon (i.e., 2–4 h), those with long duration such as the semi-triathlon distance “Ironman” (i.e., 5–8 h), and the “Ironman” distance triathlon with its 3.8 km of swimming, 180 km of cycling and 42.195 km of running (i.e., 9–16 h) and those with very long duration such as ultra-marathons or ultra-trails (some events can exceed 24 h). The effect of these PPEs on the cardiac function of participants has been the subject of much scientific research since the end of the 1990′s. The general methodology used in these various works includes the evaluation of echocardiographic parameters of the cardiac function before and after PPE under resting conditions.

After a marathon running, the majority of studies have reported a decrease in LV and right ventricular (RV) diastolic function. Interestingly, the decrease in diastolic function was effective after 1 h of exercise [7]. More recently, it has been reported that cardiac fatigue is present but with left and right ventricular dysfunction, even more marked than at rest [8]. This study underlined the importance of the intensity of exertion during a marathon in the occurrence of cardiac fatigue. In summary, a moderate duration PPE results in a decrease in LV and RV diastolic function associated with a decrease in ventricular relaxation. The results concerning LV and RV systolic function are contradictory and seem to show that the myocardial alterations are rather dependent on the intensity with which the marathon is performed.

It is important to note that the decreases in systolic function and diastolic function of LV and RV observed in the literature following various PPE have mainly been demonstrated by standard echocardiography and tissue Doppler variables. The development of speckle tracking has made it possible to go further in the evaluation of ventricular myocardial function. Thus, it was possible to assess regional myocardial deformities (e.g., apex, base), and the contractility and relaxation properties associated with these deformities and with the rates of myocardial deformation. The results concerning the studies carried out after a PPE of moderate duration are more contrasted [9,10,11,12,13]. Among these studies, only one was conducted after a marathon race. On the one hand, it is clearly established that this type of exercise leads to a decrease in LV and RV diastolic function associated with a decrease in myocardial relaxation. On the other hand, doubts remain concerning the systolic function of the LV [7] and not that of the RV, which seems to be regularly affected by the different types of PPE [14]. In fact, the left ventricular deformities in systole are reduced after exercise while the associated systolic rates are not. These findings may be due to tachycardia and higher circulating plasma catecholamine levels after EPP [15]. All these points show in particular the impact of the duration of the effort on the occurrence of cardiac fatigue in some athletes.

In this context, the aim of the present review was to provide a state of the art of the last 10 years based on published studies focusing on cardiac fatigue following a marathon. The second objective was to give an advice to athletes to continue their passion by decreasing the impact of the most intense PPE on cardiac function and structure.

## 2. Materials and Methods

This review analyzed the responses of the cardiovascular system after a marathon. The PubMed, Bookshelf and Medline databases were queried during a time span of 10 years (i.e., 1 January 2010 to 1 August 2021) using the following words: “cardiac fatigue AND marathon” and “cardiac stress AND marathon”. The PRISMA method has been used to perform this review. The inclusion criteria were: cardiovascular system structure and function evaluations (all experimental technics of analysis) performed before and immediately after a marathon (i.e., 42.2 km), and biomarkers and molecular responses to a marathon. The exclusion criteria were: all studies performed on half-marathon, on longer races (e.g., ultra-marathon), on exercises trying to mimic the duration and the intensity of a marathon (e.g., ergocycle, treadmill) and on marathons performed in extreme environments (e.g., mountain, desert). Exclusion criteria were also: (1) duplicates, (2) studies not assessing cardiac function or biomarkers before and after a marathon.

## 3. Results

### 3.1. Search Results

**Figure 1:**
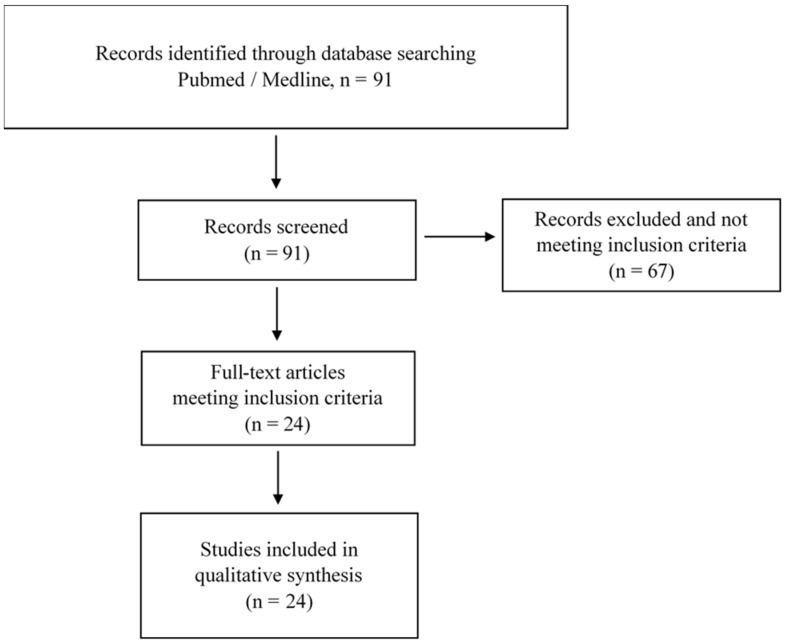
Flow chart of the published studies selection process of the review.

Figure 1 presents the selection process. To perform this review, the Pubmed, Bookshelf and Medline databases were queried and the PRISMA method was used. A total of 91 articles were identified. Following this search, duplicate references were removed. After this identification step, we proceeded to the screening step, which involved sifting through the titles and abstracts to check their relevance. Studies were selected if they were conducted only for marathons and if they studied the impact of this specific running on cardiac fatigue or cardiac stress. Ninety-one papers were selected, and their full texts were reviewed by the authors for inclusion in the review. Following the expertise of the selected articles, 24 papers were considered in the writing of the review.

### 3.2. Biomarkers of Cardiac Fatigue and Cardiac Stress after a Marathon

Sixteen papers were identified in this review and are presented in Table 1. All of them were experimental studies and investigated the change in specific biomarkers between pre- and post-marathon runs. At least 32 different biomarkers were identified in the different studies. The majority of them were biomarker of skeletal muscle and myocardium damage [15,16,17,18,19,20,21,22,23]. In this family, the creatine kinase (CK), the highly sensitive cardiac troponin I and T (hs cTnI; hs cTnT) were mainly measured in the plasma. It was demonstrated that CK and hs cTnT were significantly increased after a marathon run. A second family of biomarkers measured the cardiac injury after marathons [9,17,18,19,21,23,24,25]. The N-terminal pro brain natriuretic peptide (NT-proBNP) was mainly measured in the plasma and was significantly increased after a marathon. In addition, it was reported that the increment of this biomarker immediately after a marathon exhibited a positive curvilinear relationship (r^2^ = 0.359, *p* = 0.023) with the running time achieved by the runners [25]. A third family of biomarkers measured the systemic inflammation after marathons [23,26,27]. The interleukin-6 (IL-6) and the tumor necrosis factor-alpha (TNF-alpha) were mainly measured in the plasma. It was demonstrated that both biomarkers were significantly increased after a marathon run.

Three of the selected studies measured the heart-type fatty acid binding protein (H-FABP) (i.e., mainly found inside cardiomyocytes) after a marathon [15,18,23]. Despite an important variability between the studies, H-FABP was significantly increased after a marathon run in three studies.

In addition, two studies measured the galactin-3 (gal-3) which is a protein involved in various biological activities in different organs, including apoptotic regulation, inflammation and fibrosis [15,18]. After a marathon, this protein was significantly increased in both studies. Another two studies measured the suppression of tumorigenicity 2 (ST2) [21,28] after a marathon. They both reported a significant increase of ST2 after running. Technical issues and determination of a diagnostic threshold have to be done to fully recognize the specificity of these biomarkers.

Finally, only one study investigated the potential of circulating short nonprotein coding RNA (c-miRNA) to explore the impact of a marathon run [29]. In this study, which was conducted with 21 healthy male marathon runners, the authors demonstrated that all plasma levels of the selected c-miRNA (i.e., enriched in muscle: c-miR-1; c-miR-133a; c-miR-499-5p; enriched in myocardium: c-miR-208a; enriched in vascular endothelium: c-miR-126; marker of inflammation: c-miR-146a) were significantly increased when compared to pre-marathon. The authors also stated that these c-miRNAs might represent real-time and tissue-specific adaptation biomarkers of a marathon run.

### 3.3. Cardiovascular Function after Marathon

Seven papers were identified in this review and are presented in Table 1. All of them were experimental studies and assessed the cardiovascular function before and after a marathon run. The majority of the selected studies used echocardiography alone [9,30,31,33,35]. The majority of these studies reported a decreased E wave and/or an E/A ratio after a marathon. They also all reported no significant difference of the LV EF values between pre- and post-marathon. Three of these studies used the speckle tracking imaging technique to evaluate LV and RV strains [9,30,35]. For LV function, Sengupta et al. reported a significant decrease of the global longitudinal (≈−3% in average) and circumferential (−2% in average) strains but not in the radial plane after a marathon in recreational runners with a mean age of 41 ± 8 years [9]. In their study, Chan-Dewar et al. reported a significant decrease of the LV subepicardial radial strain (−12.3% in average) sub-endocardial circumferential strain (−3.2% in average) in male non-elite marathon runners with a mean age of 32 ± 10 years [35]. On the contrary, Lewika-Potocka et al. did not report any difference for the LV global strain between pre- and post-marathon in amateur marathon runners with a mean age of 40 ± 8 years [30]. However, these authors also analyzed the RV function and they reported a significant decrease of the RV four chambers longitudinal strain after a marathon (−1.2% in average).

Moreover, one study used the heart rate variability to assess the cardiac autonomous nervous system [32] and one study assessed cardiac function with cardiac magnetic resonance and echocardiography [34], pre- and post-marathon. In the first study, the authors reported a significant increase of the cardiac sympathetic activity (+30 min) and of the heart rate in supine position (+30 bpm) after a skyrunning marathon (i.e., 42 km distance with an ascent distance of 3.15 km and a descent distance of 2.85 km) in healthy male amateurs with a mean age of 37 ± 9 years. In the second study, the authors demonstrated a significant decrease of the LV E/A ratio and of the LV septal E’ and A’ waves after a marathon with male amateur runners with a mean age of 41 ± 5 years. In addition, they demonstrated no difference between pre- and post-marathon for the LV radial shortening and the circumferential and longitudinal strains assessed by MRI. However, the analysis revealed an increase in LV torsion and maximal torsion velocity after a marathon.

## 4. Discussion

A growing number of recreational runners are interested in pushing their limits or running for a moment next to a world champion during a marathon [6].

In this context, the present review considered 24 studies investigating the impact of marathon running, only, on cardiac fatigue or stress.

The “prototype” of the recruited participant in these studies is the following: male, aged around 35–40 years and running a marathon in 200 min or more. Thus, the message of this review is specific to this population.

The majority of the selected studies investigated a variety of biomarkers between pre- and post-marathon trying to characterize a biochemical signature of cardiac fatigue or stress in runners.

There is a clear impact of marathon on skeletal muscle and myocardium structure. Indeed, it has been reported that CK, cTnT and cTnI were increased post-marathon in the plasma of runners [15,16,17,18,19,20,21,22,23]. These plasmatic elevations suppose muscle damages after a marathon. Moreover, the NT-proBNP is also significantly increased after a marathon suggesting a potential cardiac injury in runners [9,17,18,19,21,23,24]. In addition, IL-6 and TNF-alpha were both increased after a marathon [23,27,33] suggesting an increase in inflammation induced by the race. Finally, it is noteworthy that Wilson et al., 2012, conducted one of the first studies on biomarkers after a marathon [36]. In their study, which is conducted on 25 athletes who ran the marathon in 4 h in average, the authors identified 36 proteins in the serum, which were significantly correlated with changes in the right ventricle ejection fraction after the marathon. Five proteins were identified pre-race (e.g., IL-8), 16 at the finish line (e.g., calmodulin) and 15 after 7 h post-race (e.g., serum amyloid A protein 1). Since this last part demonstrates a clear immediate negative impact of a marathon for the myocardium structure and cardiac function, other biomarkers measured pre- and post-marathon may contribute to fully explore their potential relationships with marathon-induced cardiac fatigue.

Regularly exercise is highly beneficial for individual’s health [37] and longevity [38]; however, the acute effect of PPE is, for some runners, potentially deleterious for their cardiac health [6]. This review focused on marathon running and summed up the studies investigating the impact of marathon on cardiac function in the last decades. Before 2010, it has been reported that marathon running induces a decrease in LV and RV diastolic function [7,39,40,41]. This decrease is characterized by a decrease in the E/A ratio linked to an increase in the A wave, a decrease in the E wave and overall a decrease in the E wave changes in vascular and cardiac function after prolonged strenuous exercise in humans [39,40]. The latter seems to be linked to a decrease in LV relaxation [42]. However, the results were more contrasted concerning the systolic function of the LV and the RV after a marathon. Between 90 min and 240 min of running (i.e., marathon-type efforts), the majority of studies did not report any deterioration in systolic function with unchanged or increased EF post-race [39,40,41,43]. These contradictory results can be explained by the fact that the measured parameters are not completely independent of the cardiac load conditions. In addition, increased plasma catecholamine concentration [44] post-exercise may modulate the contractile properties of LV which may improve the systolic function. The studies included in this review reinforced the point that marathon running induces a clear LV diastolic dysfunction [9,31,33,34,35]. In addition, this review also reinforced the point that a marathon did not seem to alter the LV EF but tended to increase it post-marathon [9]. This point may be explained by higher circulating plasma catecholamine post-race or by an increase in cardiac sympathetic activity (i.e., increase of the sympathovagal balance (Ln LF/HF)) [32]. To go deeper into the cardiac function assessment, a very limited number of studies investigated cardiac function post marathon by speckle tracking echocardiography. One study measured the LV diastolic and systolic functions post marathon using strains and strain rates (i.e., diastolic and systolic) analyses at the sub-endocardium and sub-epicardium level [35]. Only a significant reduction of the sub-epicardial radial strain and of the sub-endocardial circumferential strain were demonstrated showing a small regional alteration of the LV myocardium after a marathon [35]. The two other studies demonstrated no LV myocardial contractile impairment [30] or a regionalized reduction of the LV myocardial strain (longitudinal and circumferential) [9]. Only one study investigated the RV function after a marathon and demonstrated a significant reduction of the RV global strain [30]. Based on these limited data, it seems that marathon running induces a small and regionalized impairment of the LV contractile function with no evidence on the LV relaxation reduction. In contrast to this last point, the RV contractile function might be reduced after a marathon with no potential impairment of its contractility.

## 5. Conclusions

In conclusion, PPE causes, in some athletes, a transient decline in heart function. This phenomenon is often associated with an increase in biomarkers of myocardial degradation. When focusing on marathon, this review clearly demonstrated a weak impact on LV and RV systolic and contractile function and a negative impact on LV diastolic function in recreational runners. This review also pointed out the transient negative (i.e., inflammation, damage) impact of marathon running on myocardium in this population. These transient alterations demonstrate the physiological nature of cardiac fatigue induced by a marathon.

In addition, the training status [41] and the running intensity [45] influence the amplitude of cardiac function impairment and biomarkers release after a marathon. Moreover, it has recently been reported that marathon runners over 40 years old who completed a marathon between 2018 and 2019 had a 20% prevalence of coronary artery disease [46]. Based on these last elements and on the present review, it is important that marathoners and, more specifically, recreational runners adapt their training plans and perform a regular medical screening before engaging in marathon races.

Considering the growing number of amateur participants in marathons around the world, this review on the overall effect of this type of PPE on cardiac function provides information to continue to run marathons while preventing the potential cardiac risks associated with the repetition of this type of effort on the scale of an athlete’s life.

### Future Directions

Future studies in this field may be conducted on women to better understand the impact of marathons on the cardiac function in this specific population.

In addition, as the most famous marathons are performed in cities (e.g., Boston, New York, Paris) and consequently in polluted environments, future studies might focus on the impact of marathon performed in polluted but also in cold, hot and humid environments on the cardiac function.

## Figures and Tables

**Table 1:** Cardiac fatigue, cardiac stress and marathon.

References	Methods/Parameters	Pre-Marathon	Post-Marathon	*p*-Value
	Biomarkers Analyses			
Traiperm [25]				
	cTnT (ng/mL)			
	NT-proBNP (pg/mL)		Curvilinear relationship between NT-ProBNP increment and running time (r^2^ = 0.359)	<0.05
Kaleta-Duss [15]				
	CK (U/l)	148 ± 76.3	411 ± 170	<0.001
	hs-cTnI (ng/mL)	0.01 ± 0.01	0.06 ± 0.09	<0.001
	H-FABP (ng/mL)	2.22 ± 1.18	13.57 ± 9.63	<0.001
	BNP (pg/mL)	79.86 ± 53.11	155.38 ± 156.23	<0.001
	NT-proANP (pg/mL)	469.25 ± 155.44	753.3 ± 176.60	<0.001
	Gal-3 (ng/mL)	8.53 ± 3.04	10.65 ± 2.33	<0.001
	GDF-15 (pg/mL)	50.97 ± 27.61	137.34 ± 85.19	<0.001
Martinez-Navarro [16]				
	hs-cTnT (ng/L)	5.74 ± 5.29	50.4 ± 57.04	<0.001
Sierra [26]				
	IL-6 (pg/mL)	581 ± 1529	87 ± 53	NS
	IL-8 (pg/mL)	3099 ± 6511	1450 ± 6233	NS
	IL-12p40 (pg/mL)	3775 ± 12406	285 ± 131	<0.05
	IL-23 (pg/mL)	3722 ± 12115	1004 ± 254	<0.05
	IL-33 (pg/mL)	412 ± 1546	267 ± 145	<0.05
	TSLP (pg/mL)	387 ± 1974	20 ± 16	<0.05
	eNO (ppb)	20 ± 11	35 ± 19	↑
Wegberger [17]				
	Troponin I (µg/L)	btw 0–0.01	0.03 (0.02–0.05)	0.016
	CK (U/L)	btw 0–250	425 (327–681)	0.001
	Copeptin (pmol/L)	btw 0–20	26.25 (16.29–39.02)	0.078
	NT-proBNP (ng/L)	btw 0–100	132 (64–198)	0.001
	MR-proADM (nmol/L)	btw 0.25–0.60	0.88 (0.55–0.99)	0.023
de Gonzalo-Calvo [18]				
	hs-cTnT (pg/mL)	btw 0–5	btw 0–35	<0.01
	NT-proBNP (pg/mL)	btw 0–25	btw 0–110	<0.05
	CK (U/L)	btw 0–150	btw 0–300	<0.001
	hFABP (ng/mL)	btw 0–3	btw 0–24	<0.01
	Gal-3 (ng/mL)	btw 0–7	btw 0–22	<0.001
Kosowski [19]				
	hs-cTnI (pg/mL)	3.67 (1.88–5.38)	22 (9.58–34.56)	<0.001
	NT-proBNP (pg/mL)	50 (33–73)	169 (112–365)	<0.001
	ET-1 (pg/mL)	3.03 (2.5–3.4)	5.22 (4.4–5.89)	<0.001
	Creatinine (mg/dL)	0.85 (0.79–0.98)	1.39 (1.22–1.56)	<0.001
Richardson [20]				
	cTnT (ng/L)	5.60 ± 3.27	74.52 ± 30.39	<0.001
Sengupta [9]				
	NT-proBNP (pg/mL)	86.0 ± 9.5	106.5 ± 24.2	0.001
Clauss [24]				
	Chromogranin A (pg/mL)	btw 0–60	btw 0–90	<0.001
	NT-proBNP (ng/mL)	btw 0–30	btw 0–110	<0.001
Roca [21]				
	NT-proBNP (ng/L)	70 (70–70)	92 (70–147)	<0.001
	ST2 (ng/mL)	34.2 (24.7–40.9)	54.2 (38.2–72.4)	<0.001
	hs-TnT (ng/L)	2.9 (1.7–7)	46.9 (24.1–91.1)	<0.001
Bekos [28]				
	sRAGE (pg/mL)	btw 250–600	btw 400–750	<0.001
	ST2 (pg/mL)	btw 0–250	btw 125–400	<0.001
Niemelä [27]				
	suPAR (ng/mL)	btw 0.5–2	btw 1.2–3.5	<0.01
	CD163 (ng/mL)	btw 300–800	btw 500–1100	<0.05
	CRP (mg/L)	btw 0–12	btw 0–22	<0.05
	IL-6 (pg/mL)	btw 0–8	btw 17–25	<0.01
	IL-8 (pg/mL)	btw 5–12	btw 25–42	<0.05
	IL-10 (pg/mL)	btw 0–1	btw 1–3.5	<0.05
	TNF-α (pg/mL)	btw 0–1	btw 1–2.5	NS
	TGF-β (pg/mL)	btw 500–1000	btw 0–1000	NS
Martin [22]				
	Creatinine (mg/dL)	0.94 ± 0.12	1.42 ± 0.24	<0.001
	CK (U/L)	133 ± 60	367 ± 167	<0.001
	White blood cells (thousand/μL)	5.75 ± 1.19	15.77 ± 3.29	<0.001
	Neutrophils (cells/μL)	3420 ± 1049	13580 ± 3019	<0.001
Scherr [23]				
	hs-cTnT (ng/L)	3 (3–5)	31 (19–47)	<0.001
	NT-proBNP (ng/L)	27 (14–40)	93 (57–150)	<0.001
	h-FABP (Kg/L)	7 (5–10)	45 (32–64)	<0.001
	hs-CRP (mg/L)	0.52 (0.30–0.93)	0.40 (0.24–0.85)	<0.001
	IL-6 (ng/L)	2.1 (1.9–2.2)	32 (21–41)	<0.001
	IL-10 (ng/L)	5.1 (4.9–5.4)	20 (11–50)	<0.001
	TNF-α (ng/L)	9 (7–10)	10 (9–12)	<0.001
	Cystatin C (mg/L)	0.8 (0.7–0.9)	0.9 (0.9–1.0)	<0.001
Baggish [29]				
	c-miR-1 (fold change)		21.8	0.04
	c-miR-126 (fold change)		1.9	<0.001
	c-miR-133 (fold change)		18.5	0.02
	c-miR-134 (fold change)		1.9	<0.001
	c-miR-146a (fold change)		3.3	<0.001
	hsCRP (fold change)		1.0	1.000
	**Echography, HRV & STE analyses**			
Lewicka-Potocka [30]				
	LV EF (%)	61.8 ± 4.9	60.5 ± 4.4	0.38
	LV GLS (%)	−19.9 ± 2.3	−19.4 ± 2.1	0.41
	RV 4CSL (%)	−22.0 ± 2.8	−20.80 ± 2.6	<0.05
	TAPSE (mm)	25.0 ± 3.6	24.0 ± 3.7	0.56
	RVd MID (cm)	3.4 ± 0.6	3.7 ± 0.5	<0.01
	RVd BAS (cm)	3.8 ± 0.4	3.8 ± 0.5	0.44
	LVd BAS (cm)	4.8 ± 0.4	4.6 ± 0.3	<0.001
	RVd/LVd BAS	0.77 ± 0.1	0.82 ± 0.1	<0.05
Roeh [31]				
	E/A	1.6 ± 0.5	1.1 ± 0.3	<0.001
	E/e’ mean	6.4 ± 1.5	6.5 ± 1.8	0.6
	DT (s)	0.18 ± 0.05	0.20 ± 0.05	<0.001
	V_min_ (mL/m^2^)	11.4 ± 3.7	9.9 ± 3.5	<0.01
	V_max_ (mL/m^2^)	28.0 ± 6.2	25.0 ± 7.0	<0.01
	Total-SV (mL/m^2^)	59.6 ± 7.8	60.7 ± 6.0	0.3
	Total-EF (%)	34.9 ± 8.6	31.33 ± 10.2	<0.01
	ASV (mL/m^2^)	16.6 ± 3.8	15.1 ± 4.1	<0.01
	True-EF (%)	6.1 ± 2.4	4.8 ± 2.8	<0.001
Sengupta [9]				
	Heart rate (beats/minute)	74.1 ± 6.4	64.5 ± 7.6	<0.001
	Systolic BP (mmHg)	123 ± 11	120 ± 9	0.214
	Diastolic BP (mmHg)	79 ± 5	79 ± 5	0.675
	IVSd (cm)	0.94 ± 0.16	1.03 ± 0.20	0.005
	LV mass (gm)	0.94 ± 0.16	1.03 ± 0.20	0.005
	LV mass (gm)	120.2 ± 30.0	160.3 ± 43.0	<0.001
	LVEDV (mL)	61.8 ± 16.5	72.8 ± 5.1	<0.001
	LVESV (mL)	21.9 ± 7.5	20.3 ± 3.7	0.191
	LVEF (%)	64.9 ± 5.6	72.0 ± 5.7	<0.001
	Mitral E (cm/s)	89.8 ± 17.1	80.1 ± 17.0	0.001
	Mitral annular e0 (cm/s)	10.4 ± 2.1	10.1 ± 2.2	0.638
	Mitral E/e0	9.1 ± 2.4	8.3 ± 2.7	0.227
	Left atrial volume index (mL/m^2^)	23.2 ± 6.1	19.0 ± 6.5	0.01
	LV global longitudinal strain (%)	−19.3 ± 2.71	−16.5 ± 4.6	0.003
	LV global circumferential strain (%)	−17.2 ± 2.41	−15.2 ± 2.6	0.001
	LV global radial strain (%)	31.9 ± 7.4	30.9 ± 1.3	0.422
Mertová [32]				
	Sympathovagal balance	-	Ln LF/HF	↑
	Heart rate (bpm)	-	+30	
Sierra [33]				
	Peak VO_2_ (mL/kg/min)	51 (46–52)	46 (43–49)	<0.05
	Peak VE (L/min)	134 (99–148)	120 (111–147)	NS
	VE/VCO_2_ slope	34 (30–41)	31 (27–39)	<0.05
	HR	62 (60–67)	104 (101–111)	<0.05
	Systolic volume	80 (79–100)	61 (51–68)	<0.05
	Cardiac output	5354(4747–6458)	6234(5238–7433)	NS
	LVEDD	51(49–52)	51 (45–58)	NS
	LVESD	32 (29–32)	32 (28–34)	NS
	EF	67 (66–70)	62 (61–67)	NS
	E wave	0.9 (0.7–1.0)	0.6 (0.5–0.7)	<0.05
	A wave	0.7 (0.5–0.9)	0.9 (0.8–0.9)	NS
	E/A ratio	1.3 (1.1–1.5)	0.7 (0.6–0.8)	<0.05
	s’ wave	8.8 (8.2–9.7)	6.7 (5.9–8.0)	<0.05
	e’ wave	9.2 (8.4–10.6)	8.5 (6.4–10.4)	NS
	a’ wave	8.1 (7.6–9.1)	7.6 (6.6–9.6)	NS
	E/e’ ratio	0.09 (0.08–0.10)	0.08 (0.06–0.09)	NS
Hanssen [34]				
	Heart rate (beats/min)	57 ± 7	86 ± 13	<0.001
	Systolic blood pressure (mmHg)	132 ±13	121 ± 12	<0.001
	Diastolic blood pressure (mmHg)	86 ± 8	74 ± 7	<0.001
	LVEF (%)	65 ± 4	67 ± 5	0.280
	LV end-diastolic volume (cm^3^)	120 ± 25	113 ± 27	0.142
	E (cm/s)	74 ± 14	66 ± 14	0.054
	A (cm/s)	56 ± 13	72 ± 12	<0.001
	E /A ratio	1.4 ± 0.3	0.9 ± 0.2	<0.001
	Septal E’ (cm/s)	10 ± 1	8 ± 2	0.001
	Septal A’ (cm/s)	10 ± 2	12 ± 3	0.001
	E /E’ ratio	8.3 ± 1.6	8.4 ± 3.4	0.871
Chan-Dewar [35]				
	Sub-epicardial radial strain (%)	32.6 ± 12.5	20.3 ± 9.6%	<0.01
	Sub-endocardial circumferential strain (%)	−26.9 ± 3.6	−23.7 ± 4.1	<0.01
	EF	63 ± 5	62 ± 7	NS
	E/A	1.8 ± 0.7	1.1 ± 0.2	<0.01

4CSL: four-chamber longitudinal strain = global strain; ASV: atrial stroke volume; BNP: B-type natriuretic peptide; BP: blood pressure; Bpm: beats per minutes; Btw: between; CK: creatine kinase; DT: deceleration time; E: early diastolic mitral inflow velocity; E/e’: ratio of early diastolic mitral inflow to mitral annular velocity; e’: early diastolic mitral annular velocity; EDD: end-diastolic diameter; EF: ejection fraction; eNo: exhaled nitric oxide; ESD: end-systolic diameter; ESV: end-systolic volume; FAC: fractional area change; Gal-3: galectin 3; GDF-15: growth differentiation factor 15; GLS: global longitudinal strain; H-FABP: heart-type fatty acid binding protein; HRV: heart rate variability; hs-cTnI: high sensitivity cardiac troponin I; IL: interleukin; IVSd: diastolic interventricular septum thickness; LF/HF: low-frequency power/high-frequency power; LV: left ventricle; LVd BAS: LV basal end-diastolic diameter; LVEDD: left ventricular end-diastolic diameter; LVEDV: left ventricular end-diastolic volume; LVEF: left ventricular ejection fraction; LVESD: left ventricular end-systolic diameter; LVESV: left ventricular end-systolic volume; NT-proANP: N-terminal proatrial natriuretic peptide; PWd: posterior wall in diastole; PWs: posterior wall in systole; RV: right ventricle; RVd BAS: RV basal end-diastolic diameter; RVd MID: RV mid-cavity end-diastolic dimension; RVd/LVd BAS: basal RV to LV end-diastolic diameter ratio; S: peak systolic pulmonary venous flow velocity; STE: speckle tracking echography; SV: stroke volume; TAPSE: tricuspid annular plane systolic excursion; Total-EF: total ejection fraction; Total-SV: total stroke volume; True-EF: true ejection fraction. Data are expressed as means, medians and interquartile ranges (25th percentile; 75th percentile) and R-squared.

## Data Availability

Not relevant for this manuscript.
